# Prophylaxis in older Canadian adults with hemophilia A: lessons and more questions

**DOI:** 10.1186/s12878-015-0022-8

**Published:** 2015-02-14

**Authors:** Shannon C Jackson, Ming Yang, Leonard Minuk, Michelle Sholzberg, Jean St-Louis, Alfonso Iorio, Robert Card, Man-Chiu Poon

**Affiliations:** Division of Hematology, Department of Medicine, University of British Columbia, Vancouver, BC Canada; British Columbia Provincial Bleeding Disorders Program – Adult Division, 1081 Burrard Street, Comox Building, Room 217, Vancouver, British Columbia V6Z 1Y6 Canada; Schulich School of Medicine and Dentistry, University of Western Ontario, London, ON Canada; St Michael’s Hospital, Toronto, ON Canada; CHU-Sainte-Justine and Department of Medicine, University of Montreal, Montreal, QC Canada; Clinical Epidemiology & Biostatistics and Medicine, McMaster University, Hamilton, ON Canada; Division of Hematology, Department of Medicine, University of Saskatchewan, Saskatoon, SK Canada; Division of Hematology and Hematologic Malignancies, Department of Medicine, University of Calgary, Calgary, AB Canada

**Keywords:** Hemophilia A, Adults, Prophylaxis, Bleeding, Aging, Factor VIII

## Abstract

**Background:**

Although prophylaxis is a standard of care for young children in developed countries, known to reduce the severity of hemophilic arthropathy, older adults with existing arthropathy have not traditionally used prophylaxis. Recent studies have shown that adults with hemophilia A are increasingly adopting prophylaxis but the characteristics of this treatment in older adults are not well understood. This multicenter observational study was conducted to describe how secondary/tertiary prophylaxis is being used in older adults (≥40 years of age) in comparison to younger adults with severe hemophilia A.

**Methods:**

Eligible adult (≥18 years of age) Canadian males with baseline FVIII:C ≤2% from the participating centres were observed over a 2 year period.

**Results:**

Of the 220 adult severe hemophilia patients enrolled, 70% (155/220) used prophylaxis during the observational period. Only 27% (60/220) are older adults with very few >60 years of age. A lower proportion of older adults use prophylaxis compared to younger adults (58% vs. 75%, p = 0.016), with most patients in both groups using continuous prophylaxis (92 and 94% respectively). When considering all treatment modalities together, younger subjects use more factor concentrate than older subjects (2437 u/kg/year vs. 1702 u/kg/year, p = 0.027); however, older subjects on prophylaxis use 3447 u/kg/year and had an ABR of 12 while those on demand use 560 u/kg/year and had an ABR of 13.

**Conclusion:**

A significant number of older adults use secondary/tertiary continuous prophylaxis in Canada, accounting for a significant fraction of factor concentrate utilization.

## Background

Prophylaxis, “treatment by intravenous injection of factor concentrate in anticipation of and in order to prevent bleeding”, is a contemporary standard of care for children and adolescents with severe hemophilia A in the developed world [1]. When initiated in the absence of documented osteochondral joint disease, and before the second clinically evident large joint bleed (primary prophylaxis) [2], benefits include: a reduction in number of spontaneous joint and muscle bleeds, prevention or slowing of hemophilia related joint disease, reduced hospitalization rates and times, improved school performance and increased quality of life. [3,4]. Early prophylaxis has allowed boys with hemophilia in developed countries to live with substantially less hemophilic arthropathy and disability than past generations.

In Canada, individuals with moderate or severe hemophilia A older than 40 years often live with significant joint disease from recurrent joint bleeds because prophylaxis was not available until they were already adults. For the most part, prophylaxis in adults was adopted after 1987 when later generation virally inactivated plasma-derived, followed by recombinant, factor concentrates were available assuring both safety from blood-borne infection and supply.

With the increasing use of prophylaxis, new definitions have emerged that describe 2 forms of prophylaxis that are distinct from primary prophylaxis. Secondary prophylaxis (previously designated early secondary prophylaxis) is the regular continuous treatment started after 2 or more joint bleeds (but before the onset of joint disease detectable by physical examination or imaging studies) and tertiary prophylaxis (previously designated late secondary prophylaxis) is started after the onset of joint disease [2]. Recent evidence has suggested that, in spite of existing joint arthropathy, tertiary prophylaxis offers benefit to adults including decreased annualized bleeding rate (ABR), less missed days of school/work and improved quality of life [5-7]. A Canadian survey conducted in 2006 estimated that 55% of adults with severe hemophilia A ≥ 18 years of age, including 40% of those >50 years of age, were on prophylaxis. On this background, we hypothesized that prophylaxis in both age groups is increasing in Canada [8]. However, inherent differences in the older (≥40 years of age) group who were not exposed to primary prophylaxis in childhood resulting in a great burden of joint arthropathy and co-infections, may alter how prophylaxis is used when compared to the younger age group. Therefore, a study was conducted to describe how secondary/tertiary prophylaxis is being used in older adults (≥40 years of age) in comparison to younger adults with severe hemophilia A. Characterizing this older age group is important because their number will steadily increase until those exposed to primary prophylaxis for most of childhood reach this phase of life.

## Methods

Canadian adult males, ≥18 years of age with severe hemophilia A (FVIII:C ≤2%), from 7 centres were included after institutional review board approval from the following ethics boards: University of British Columbia/Providence Health Care Research Institute, University of Calgary, University of Saskatchewan, University of Western Ontario, McMaster University, University of Toronto and University of Montreal. Inclusion criteria was intended to allow those patients with both <1% and 1-2% FVIII who use prophylaxis to be characterized because of the phenotypic overlap between moderately severe and severe hemophilia A. Data was extracted retrospectively from the medical chart and/or electronic medical record and the local Canadian Hemophilia Assessment and Resource Management System (CHARMS, http://ahcdc.ca/index.php/charms) [9], with a standardized case report form at each site. CHARMS was the primary source for factor utilization data across centres and all utilization data was extracted locally. Some centres use the system as a local electronic medical record. The observation period was from July 1, 2009 to June 30, 2011 (2 years). The local medical chart and/or electronic medical record was considered the primary source of information related to the use of prophylaxis.

Prophylaxis was defined as the use of infused factor concentrate to prevent anticipated bleeding. Duration of exposure was defined as either continuous with ≥ 45 weeks of receiving prophylaxis, with the intent of treating 52 weeks per year, or intermittent (periodic) where the treatment period did not exceed 45 weeks in a year, as per the World Federation of Hemophilia definitions [2]. Intermittent prophylaxis was further defined as short term (4–11 weeks/year), where the intent is usually to temporarily interrupt the bleeding cycle, or intermediate (12–44 weeks/year). Information was also gathered on whether the prophylaxis regimen was stable or variable (including with changes in dose or frequency, stopping or starting) during the observation period.

Baseline data including type of hemophilia, baseline factor VIII level, inhibitor history, number of joints affected, prior experience with prophylaxis, co-infections, and yearly factor concentrate utilization was gathered from the clinic chart prior to July 1, 2009. All available data related to prior prophylaxis exposures before 2009 was also collected. Bleed rate data was extracted from patient-submitted paper or electronic bleed logs and factor VIII utilization extracted from the CHARMS database, including factor utilization for 5 years prior to July 1, 2009 (‘pre-observation period’).

Subjects were excluded if less than 6 months of data was available in the pre-observation period and/or if less than 6 months of data was available during the 2 year observation period. Data collected during the observation period included type of treatment (on-demand or prophylaxis), duration of exposure to prophylaxis, dose and frequency used, stability of dosage regimen, reasons for starting, stopping or changing doses (when available), number of bleeds and yearly factor concentrate consumption.

Comparison of baseline demographic characteristics between age groups and between baseline FVIII groups was performed using Wilcoxon rank-sum test, chi-squared test or Fisher’s Exact test as appropriate. The univariate relationship between the outcome of prophylaxis exposure and clinical variables potentially associated with prophylaxis exposure in older subjects and younger subjects were examined using Fisher’s exact test or Chi square test or Wilcoxon rank-sum test as appropriate. Using multivariate logistic regression analysis, we identify patient or hemophilia-related factors associated with using prophylaxis during the observation period in all subjects and in each age group. A stepwise regression technique based on goodness of fit (AIC) was employed to determine the set of variables that are most predictive on the outcome. Linear regression analysis was used to examine the relationship of factor utilization during study period with age and annualized bleeding rate.

## Results

### Baseline characteristics

220 subjects with baseline FVIII ≤ 2% were included representing 84% of the eligible population in participating centres. Treatment centres ranged in size of the severe hemophilia A population (9–74 patients) but there was no correlation observed between center size and enrollment rate (Kendall’s correlation = 0.048, p = 1). The study includes 32% (165/509) of the Canadian adult hemophilia A population with baseline FVIII:C ≤1% [10]. Subjects with 1-2% FVIII:C activity at baseline represented 25% of the population (n = 55). Median (IQR) age was 30.6 years (23.7; 41.6) with an age span of 18–74 years. Baseline comparison between younger subjects (18–39 years of age) and older subjects (40+ years of age), who would not have had exposure to prophylaxis as children, are shown in Table 1. As anticipated, older subjects had a higher total number of joints with hemophilic arthropathy, a history of more joint procedures (including arthroplasty, fusion and radiosynovectomy), and a higher frequency of viral co-infections with the exception of hepatitis B. In addition to these differences, the rate of current inhibitors was higher in the older subjects at 10% versus 1% in those younger than 40 years of age (p = 0.002). Finally, the median body weight of younger subjects was 8 kg (17.6 pounds) higher than older subjects (p < 0.05). Prior to the study period, the percentage of lifetime on secondary/tertiary prophylaxis was 4% and 20% for older and younger age groups respectively. Therefore, both age groups most likely had established joint disease prior to the historical initiation of prophylaxis consistent with the definition of tertiary prophylaxis.

**Table 1:** **Population demographics by age group**

	**18-39 years**	**≥40 years**	**p value**
**Number, %**	160 (73)	60 (27)	-
**Median age (IQR), yrs**	26.8 (21.9;32.4)	48.3 (43.7;52.6)	-
**Baseline FVIII:C, no. (%)**			
<1%	119 (74)	46 (77)	NS
1%	25 (16)	10 (17)	NS
2%	16 (10)	4 (6)	NS
**Time records available**			
Median (IQR), yrs	11.2 (4;19)	18.7 (11.1;28.8)	<0.001
Range, yrs	0.5-38	0.5-48	
**Inhibitor status no. (%)** ^**1**^			
Historical*	23 (15)	15 (25)	NS
Current	2 (1)	6 (10)	0.002
**Joints affected, median no. (%)**			
0-1	79 (49)	15 (25)	<0.001
2-4	73 (46)	31 (52)	<0.001
>4	8 (5)	14 (23)	<0.001
**Prior Joint procedures, no.**			
Median	0 (0;1)	1 (0;3)	<0.001
Range	0-4	0-8	
**Co-infections, no. (%)**			
HIV	40 (25)	35 (58)	<0.001
HCV Ab	89 (56)	48 (80)	0.001
HCV RNA+	50 (31)	31 (52)	0.005
HIV + HCV Ab	36 (23)	29 (48)	<0.001
HIV + HCV RNA+	27 (17)	22 (37)	0.002
HBSAg +	11 (7)	4 (7)	NS
**Median weight (IQR), kg**	79.5 (69.5;88.5)	71.5 (63.5;79)	0.002

^1^Data missing for 2 subjects. *Includes transient and current inhibitors.

### Prophylaxis exposure during observation period

**Figure 1:**
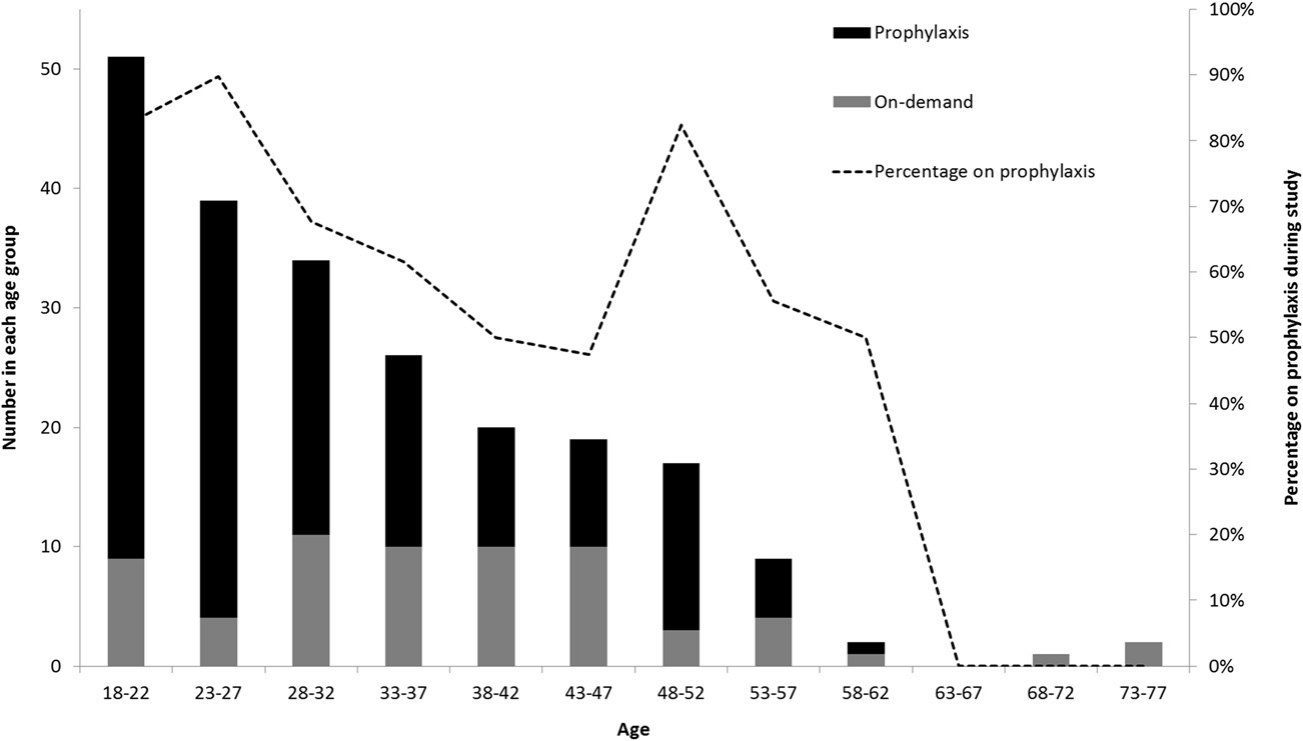
**Rate of prophylaxis exposure over the age spectrum.**

The primary outcome of this study revealed that 70% of subjects were exposed to prophylaxis during the 2 year observation period and that older individuals were less likely to use prophylaxis than younger patients (58% vs. 75%, p = 0.016), as shown in Table 2. 82-90% of the youngest adults (18–27 years) were exposed to prophylaxis with a gradual decrease in the rate with age until an abrupt increase back up to 82% was observed in subjects 48–52 years (Figure 1). This is partially accounted for by 3 individuals from that age group starting prophylaxis during the study period who were not undergoing orthopedic or other surgery when prophylaxis was started. Continuous prophylaxis (≥45 weeks of receiving prophylaxis, with the intent of treating 52 weeks per year) was used in 94% of those using prophylaxis during the observation period and did not differ between the age groups. The prophylaxis rate for subjects with baseline FVIII:C 1-2% was 62% compared to 73% for subjects with FVIII:C <1%.

**Table 2:** **Use of prophylaxis during observation period**

	**All subjects (n = 220)**	**18-39 years (n = 160)**	**≥40 years (n = 60)**	**p value**
**Prophylaxis, No (%)**	155 (70)	120 (75)	35 (58)	0.0202
Stable regimen	103 (66)	77 (64)	26 (74)	NS
Variable regimen:	52 (34)	43 (36)	9 (26)	NS
Started	11 (21)	8 (19)	3* (33)	NS
Changed	41 (79)	34 (79)	7 (78)	NS
Discontinued	11 (21)	7 (21)	2 (22)	NS
**Type of prophylaxis** ^**1**^				
Continuous	144 (94)	112 (94)	32 (92)	NS
Intermittent:	10 (6)	7 (6)	3 (8)	NS
Short-term	3 (2)	1 (1)	2 (6)	
Intermediate	7 (5)	6 (5)	1 (3)	

*All from the age 48–52 year age group.

^1^Data missing for 1 subject.

### Stable prophylaxis

60% of subjects maintained a stable prophylaxis regimen (no changes in dose or frequency) over the observation period (Table 2). For those subjects, a median dose of 2000 units (range 500–4000 units) corresponding to 25 u/kg/dose (range 6–64 u/kg/dose) was used. Older adults used higher median weight-based doses when compared to younger adults (26.8 vs. 24.6 u/kg/dose, p < 0.05) but there was no statistical difference in median factor concentrate utilization per year between older and younger adults on a stable regimen. The frequencies used were 3x weekly (40%), 2x weekly (20%), every other day (12%), daily (9%), 1x weekly (6%) and other/unknown (13%).

### Variable prophylaxis

In those with variable regimens during the observation period, the numbers who changed or stopped prophylaxis remained consistent over the age spectrum. It was not possible to compare age groups with respect to reasons for starting, stopping or changing due to small numbers in each subgroup. In those who started prophylaxis during the observation period the most common reason, cited in 67% of cases, was frequent bleeding and/or increased activity. For those who discontinued prophylaxis, the most common reason was patient desire because of time required/inconvenience (36%), difficulty with IV access (10%), patient desire for other reasons (20%) and in 36% discontinuation was documented without a reason. In those changing the prophylaxis regimen, the reasons included the health care professional recommending change (21%), anticipated or actual increase in physical activity (20%), frequent bleeding episodes (12%) and patient request due to IV access or other reasons (12%). Changes were made in the minority of patients due to target joint, major bleeding episode, orthopedic procedure, starting anticoagulation, clinical improvement or other. In those subjects who changed prophylaxis regimen, the most common initial regimen was a median dose of 1500 units (21.4 u/kg) given every other day.

### Factors influencing prophylaxis

In the multivariate regression model including all subjects, younger age was associated with an increased probability of using prophylaxis, with an odds ratio of 1.04 per year of age decrease (95% CI, 1.01 to 1.07; p = 0.020). This was also seen in the multivariate analysis of the 18–39 year old age group with an OR of 1.25 (95% CI, 1.10-1.41; p = 0.004), but not in the older age group, which was consistent with data presented in Figure 1. None of the other factors including baseline factor activity, number of joints affected, number of prior joint procedures, coinfections, history of inhibitor and pre-study weight were significantly correlated with the odds of using prophylaxis in the all subjects analysis and in the 18–39 age group analysis. However, pre-study yearly factor consumption (per 10,000 u increase) did correlate with increased odds of using prophylaxis, with an OR of 1.11 for all subjects (95% CI, 1.06 to 1.15; p < 0.001) and 1.36 for the 18–39 age group (95% CI, 1.21 to 1.53; p < 0.001). In the older age group, only a history of inhibitor (either current or history of transient inhibitor) was identified to have a significant negative influence on the use of prophylaxis with an OR of 0.23 (95% CI, 0.06 to 0.83; p = 0.025).

### Prophylaxis rate by treatment centre

The rate of prophylaxis use during the study period among the 7 centres ranged from 45-95% distributed as follows: <50% on prophylaxis (1 centre), 50-70% (2 centres), 71-90% (3 centres) and >90% (1 centre). Size of clinic and local study inclusion rate did not correlate with the rate of prophylaxis (Kendall’s correlation = 0.143 and −0.238 respectively; p = 0.773 and 0.562).

### Factor utilization

**Figure 2:**
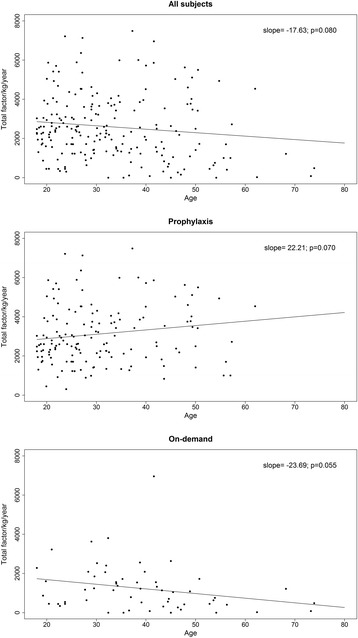
**Annual factor utilization by age based for subjects exposed to prophylaxis and on-demand treatment.**

Factor utilization data was available for 94% (206/220) of the cohort during the 2 year observation period and for 93% (205/220) for the pre-observation period (5 years prior). Factor utilization comprised total usage including that used for prophylaxis and/or bleeds. Pre-observation factor utilization (median u/kg/year) was 2057 u/kg/year (IQR 1239, 3338) and similar in the pre-observation period among all ages (Table 3). However, during the observation period those ≥40 years of age appeared to use less factor overall than younger subjects (1702 vs. 2437 u/kg/year, p = 0.027). When utilization data from subjects with current inhibitor (n = 8) were excluded this difference was no longer significant (1824 vs. 2437 u/kg/year, p = 0.120). Relationship between factor consumption (u/kg/year) and age is shown in Figure 2 with an observed trend towards decreasing overall factor utilization with age, particularly in the on-demand group. However, for those on prophylaxis, a trend towards increasing factor utilization with age was also observed but there were only a small number of older subjects. In the ≥ 40 year age group, the largest discrepancy between factor utilization for on-demand treatment versus prophylaxis was observed (560 vs. 3447 u/kg/yr, p < 0.001), and this difference remained significant when subjects with current inhibitor were excluded.

**Table 3:** **Median (IQR) factor utilization (u/kg/year) before and during observation period**

	**All subjects (n = 206)**	**18-39 years (n = 151)**	**≥40 years (n = 55)**	**p value**
**Before**	2057 (1239;3338)	2035 (1239;3343)	2143 (1200;3307)	NS
**During**	2341 (1336;3650)	2437 (1601;3650)	1702 (630;3755)	0.027
Prophylaxis	2707 (2049;3989)	2625 (2065;3857)	3447 (1931;4938)	
On-demand	1066 (446;1718)	1371 (545;2087)	560 (266;1139)	
(p value)	(<0.001)	(<0.001)	(<0.001)

### Annualized bleeding rate

**Figure 3:**
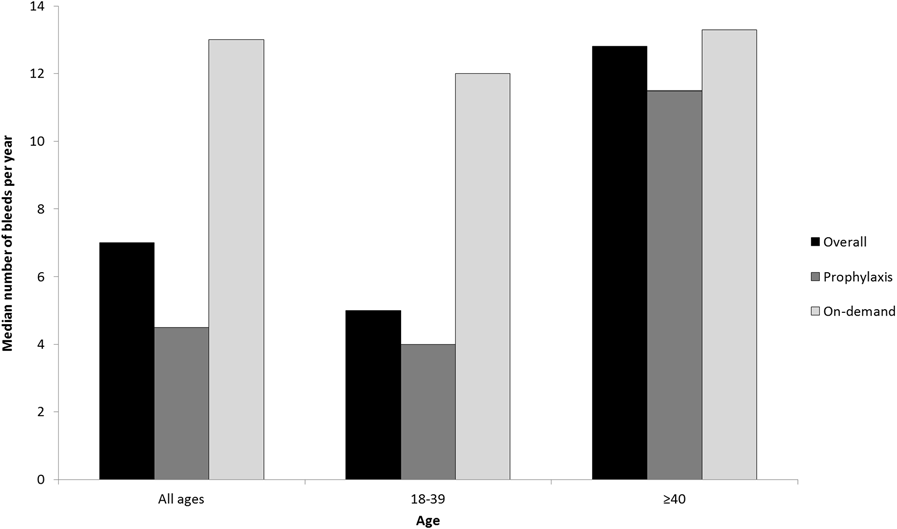
**Annualized bleeding rate by age.**

**Figure 4:**
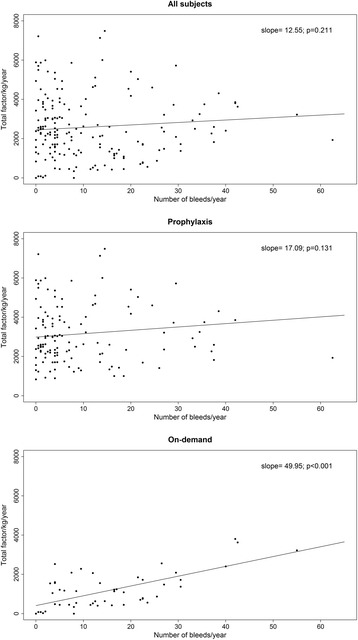
**Annual factor utilization by annualized bleeding rate for subjects exposed to prophylaxis and on-demand.**

Bleeding log data during the observation period was available for 87% (191/220) of the cohort. The median (IQR) overall ABR during the study period was 7 (3;18) bleeds/year (Figure 3). Those using prophylaxis had a lower ABR when compared to on-demand (5 vs. 13 bleeds/year, p = 0.001). Older subjects had a higher ABR than younger subjects (13 vs. 5 bleeds/year, p = 0.028) but did not exhibit a difference in ABR between prophylaxis and on-demand use (12 vs 13, p = 0.866), in contrast to the younger group (4 vs 12, p < 0.001). If ABR data from subjects with current inhibitor were excluded, prophylaxis and on-demand ABR’s in the older group remained similar (12 vs 16, p = 0.558) and ABR was unchanged in the younger group. When the relationship between factor utilization and ABR was examined, a positive correlation between ABR and factor utilization was observed only in those using on-demand treatment (Figure 4).

## Discussion

We have observed that 58% of older adults with severe hemophilia A, who would not have had exposure to prophylaxis until adulthood, are using long-term prophylaxis in Canada. This is a higher rate than previously reported in the 2007 Canadian prophylaxis survey [8], where 40% of adults > 50 years of age were using some form of prophylaxis, and a subsequent 2010 US survey where prophylaxis was used in 44% of adults 45–64 years of age (n = 454) and 32% of adults ≥65 years of age (n = 68) [11]. The definition of prophylaxis used in both surveys was similar to those used in this observational study. An increased prophylaxis rate in the younger adults was also observed here in comparison to other previously published adult North American cohorts [12-14] which suggests that the use of prophylaxis is increasing over time in both younger and older adults.

An evolving preference for prophylaxis over on-demand therapy by both patients and health care professionals may be the reason for this observation in a country with unlimited access to factor concentrates. In Canada, while prophylaxis in children is widely accepted [8,13] there are no formal guidelines to promote prophylaxis use in adults. However the United Kingdom Hemophilia Centre Doctors’ Organization (UKHCDO) and Medical and Scientific Advisory Council (MASAC) to the National Hemophilia Federation in the US (http://www.hemophilia.org/Researchers-Healthcare-Providers/Medical-and-Scientific-Advisory-Council-MASAC/MASAC-Recommendations/MASAC-Recommendation-Concerning-Prophylaxis), have both endorsed that prophylaxis be considered (or continued) in adults [15,16]. It has been demonstrated that bleeding frequency decreases significantly in adults who adopt long-term secondary prophylaxis after on-demand treatment, with reported bleed rates down to a median of 0–1 bleed per year, under study conditions [5,17,18] although only one study included adults > 50 years of age [18]. Increasing awareness of this, along with other factors such as improved quality of life, may be influencing the older adults to accept a relatively new habit of regularly self-injecting factor concentrate.

In the older cohort of the population we studied, it was surprising to observe the ABR for those using on-demand was similar to those using prophylaxis (12 vs 13 bleeds/year respectively). Furthermore, older adults used higher weight based prophylaxis doses than younger adults (26.8 vs. 24.6 u/kg/dose) but experienced almost 3-fold more bleeds annually. This finding contradicts the usual observation that adults with severe hemophilia tend to bleed less frequently than children, presumptively on the basis of lower levels of physical activity. A possible explanation, particularly plausible in the observational setting, is that those older patients on prophylaxis originally had a much higher bleeding rate, which might have prompted their decision to start prophylaxis. Unfortunately, we cannot prove or deny this hypothesis because of the incompleteness of the bleeding logs before the study. Since the majority of older men with severe hemophilia will have at least 1 joint affected with chronic hemophilic arthritis (83% in this cohort), and may have concurrent age-related osteoarthritis, there is also potential for intermittent or chronic pain to be misinterpreted as bleeding. This has been suggested by a recent study, using point of care ultrasound, where the patient perceived etiology of acute musculoskeletal pain in adults with hemophilia was correct in only 1/3 of cases [19].

A variety of patient and hemophilia specific variables were considered as possible predictors of prophylaxis use in both older and younger groups. As expected, at least in the younger group, younger age was independently associated with prophylaxis exposure but not other plausible factors such as baseline FVIII:C activity, number of joints affected with arthropathy, prior joint procedures, weight or co-infections. Factors not reflected in the medical record might also exert influence over who uses prophylaxis in both age groups including patient personality, attitudes, lifestyle and vocation, relationship with treatment centre, and inclination to follow treatment recommendations. Likewise, treatment centre nurses and physicians make their own opinion about who may benefit and this would influence treatment decisions, which is reflected in the observed variation of prophylaxis exposure between centres.

Factor concentrate supply in Canada is widely available through the medical system and affordability of factor concentrates is not an issue for patients. Yet, Canadian treaters appear to be conscientious of cost and conservative with respect to dosing. Overall annual factor utilization for prophylaxis was observed to be 2707 u/kg/year in this cohort, significantly lower than in Sweden, where FVIII utilization generally reported at ≥4000 u/kg/year [20], but higher than that reported by the Dutch groups (~2100-2500 u/kg/year) [21]. However, the discrepancy in factor utilization for prophylaxis by age group was quite surprising with the older subjects on prophylaxis using quite high amounts of factor and still exhibiting an unacceptably high ABR. A prospective study with adjudication of bleeding events is needed to follow up on this observation. However, a prospectively measured ABR collected under the rigor of a clinical study may not reflect the reality of measuring outcomes in ‘real life’ using bleed logs.

The major limitations of this study include the reliance on the medical record to reflect prophylaxis exposure rather than using data derived from patient home infusion records which could theoretically reflect actual exposure and adherence to prophylaxis. We could not accurately and confidently reconcile the annual bleed data into joint and non-joint bleeds nor provide joint scores for the population. These and other limitations of an observational study are acknowledged however several intriguing questions about the older population and their use of prophylaxis are raised. Why do older individuals use prophylaxis if the overall rate of bleeding is not different (at a population level) from those using on-demand? Why do older individuals bother with this time intensive therapy and is the benefit of prophylaxis measurable in this age group? Do adults ≥ 40 years of age really bleed more than younger adults? Given the current hemophilia guidelines that advocate to ‘treat with factor first’ an acutely painful and/or swollen joint, how do hemophilia treaters help older patients to differentiate a joint bleed from other causes of acute joint pain? Finally, is this costly treatment sustainable for the long-term with a growing number of aging patients with severe hemophilia? An understanding of the unique characteristics of a small population of older individuals sets the stage for future work to identify older patients who stand to benefit from this intensive and expensive form of treatment.

## Conclusions

In this observational study, a significant number of older individuals (≥40 years of age) with severe hemophilia were using longterm continuous prophylaxis in Canada. Weight based factor dosing and annual factor utilization for prophylaxis was higher in this older group than in younger individuals who had lower observed annual bleeding rates. Further study to explore the optimal use of prophylaxis in older individuals is warranted.

## References

[1] Iorio A, Marchesini E, Marcucci M, Stobart K, Chan AK (2011). Clotting factor concentrates given to prevent bleeding and bleeding- related complications in people with hemophilia A or B. Cochrane Database Syst Rev.

[2] Srivastava A, Brewer AK, Mauser-Bunschoten EP, Key NS, Kitchen S, Llinas A, Ludlam CA, Mahlangu JN, Mulder K, Poon M-C, Street A (2013). Guidelines for the management of hemophilia. Haemophilia.

[3] Manco-Johnson MJ, Abshire TC, Shapiro A, Riske B, Hacker M, Kilcoyne RF, Ingram D, Manco-Johnson ML, Funk S, Jacobson L (2007). Prophylaxis versus Episodic Treatment to Prevent Joint Disease in Boys with Severe Hemophilia. NEJM.

[4] Carcao MD, Aledort L (2004). Prophylactic factor replacement in hemophilia. Blood Rev.

[5] Collins P, Faradji A, Morfini M, Enriquez MM, Schwartz L (2010). Efficacy and safety of secondary prophylactic vs. on-demand sucrose-formulated recombinant factor VIII treatment in adults with severe hemophilia A: results from a 13-month crossover study. J Thromb Haemost.

[6] Tagliaferri A, Franchini M, Coppola A, Rivolta GF, Santoro C, Rossetti G, Feola G, Zanon E, Dragani A, Iannaccaro P (2008). Effects of secondary prophylaxis started in adolescent and adult haemophiliacs. Haemophilia.

[7] Tagliaferri A, Rivolta GF, Rossetti G, Pattacini C, Gandini G, Franchini M (2006). Experience of secondary prophylaxis in 20 adolescent and adult Italian hemophiliacs. Thromb Haemost.

[8] Biss TT, Chan AK, Blanchette VS, Iwenofu LN, McLimont M, Carcao MD (2008). The use of prophylaxis in 2663 children and adults with haemophilia: results of the 2006 Canadian national haemophilia prophylaxis survey. Haemophilia.

[9] Canadian Hemophilia Assessment and Resource Management System. http://ahcdc.ca/index.php/charms.

[10] Canadian Hemophilia Registry. http://www.fhs.mcmaster.ca/chr/. Accessed February 1, 2014.

[11] Zappa S, McDaniel M, Marandola J, Allen G (2012). Treatment trends for haemophilia A and haemophilia B in the United States: results from the 2010 practice patterns survey. Haemophilia.

[12] Siboni SM, Mannucci PM, Gringeri A, Franchini M, Tagliaferri A, Ferretti M, Tradati FC, Santagostino E, von Mackensen S (2009). Health status and quality of life of elderly persons with severe hemophilia born before the advent of modern replacement therapy. J Thromb Haemost.

[13] Teitel J, Belliveau D, Blanchette V, Chan A, Klaassen R, Price V, Ritchie B, Wu J (2012). A Canadian survey of self-infusion practices in persons with haemophilia A. Haemophilia.

[14] Walsh CE, Valentino LA (2009). Factor VIII prophylaxis for adult patients with severe haemophilia A: results of a US survey of attitudes and practices. Haemophilia.

[15] MASAC Recommendation Concerning Prophylaxis. National Hemophilia Foundation. New York, New York, USA; 2007.

[16] Richards M, Williams M, Chalmers E, Liesner R, Collins P, Vidler V, Hanley J (2010). A United Kingdom Haemophilia Centre Doctors’ Organization guideline approved by the British Committee for Standards in Haematology: guideline on the use of prophylactic factor VIII concentrate in children and adults with severe haemophilia A. Br J Haematol.

[17] Manco-Johnson MJ, Kempton CL, Reding MT, Lissitchkov T, Goranov S, Gercheva L, Rusen L, Ghinea M, Uscatescu V, Rescia V, Hong W (2013). Randomized, controlled, parallel-group trial of routine prophylaxis vs. on-demand treatment with sucrose-formulated recombinant factor VIII in adults with severe hemophilia A (SPINART). J Thromb Haemost.

[18] Valentino LA, Mamonov V, Hellmann A, Quon DV, Chybicka A, Schroth P, Patrone L, Wong WY (2012). A randomized comparison of two prophylaxis regimens and a paired comparison of on-demand and prophylaxis treatments in hemophilia A management. J Thromb Haemost.

[19] Ceponis A, Wong-Sefidan I, Glass CS, von Drygalski A (2013). Rapid musculoskeletal ultrasound for painful episodes in adult haemophilia patients. Haemophilia.

[20] Khawaji M, Astermark J, Berntorp E (2013). Lifelong prophylaxis in a large cohort of adult patients with severe haemophilia: a beneficial effect on orthopedic outcome and quality of life. European J Haematol.

[21] Fischer K, Carlsson KS, Petrini P, Molmstrom M, Ljung RC, Van den Berg HM, Berntorp E (2013). Intermediate-dose versus high dose prophylaxis for severe hemophilia: comparing outcome and costs since the 1970s. Blood.

